# A Smartphone-Based Decision Support Tool for Predicting Patients at Risk of Chemotherapy-Induced Nausea and Vomiting: Retrospective Study on App Development Using Decision Tree Induction

**DOI:** 10.2196/27024

**Published:** 2021-12-02

**Authors:** Abu Saleh Mohammad Mosa, Md Kamruz Zaman Rana, Humayera Islam, A K M Mosharraf Hossain, Illhoi Yoo

**Affiliations:** 1 Health Management and Informatics University of Missouri School of Medicine Columbia, MO United States; 2 Institute for Data Science and Informatics University of Missouri Columbia, MO United States; 3 Electrical Engineering and Computer Science University of Missouri Columbia, MO United States; 4 Center for Biomedical Informatics University of Missouri School of Medicine Columbia, MO United States; 5 Institute of Statistical Research and Training University of Dhaka Dhaka Bangladesh; 6 Ellis Fischel Cancer Center University of Missouri School of Medicine Columbia, MO United States; 7 Department of Hematology and Medical Oncology BayCare Health System South Florida Baptist Hospital Plant City, FL United States

**Keywords:** chemotherapy, CINV risk factors, data mining, prediction, decision trees, clinical decision support, smartphone app

## Abstract

**Background:**

Chemotherapy-induced nausea and vomiting (CINV) are the two most frightful and unpleasant side effects of chemotherapy. CINV is accountable for poor treatment outcomes, treatment failure, or even death. It can affect patients' overall quality of life, leading to many social, economic, and clinical consequences.

**Objective:**

This study compared the performances of different data mining models for predicting the risk of CINV among the patients and developed a smartphone app for clinical decision support to recommend the risk of CINV at the point of care.

**Methods:**

Data were collected by retrospective record review from the electronic medical records used at the University of Missouri Ellis Fischel Cancer Center. Patients who received chemotherapy and standard antiemetics at the oncology outpatient service from June 1, 2010, to July 31, 2012, were included in the study. There were six independent data sets of patients based on emetogenicity (low, moderate, and high) and two phases of CINV (acute and delayed). A total of 14 risk factors of CINV were chosen for data mining. For our study, we used five popular data mining algorithms: (1) naive Bayes algorithm, (2) logistic regression classifier, (3) neural network, (4) support vector machine (using sequential minimal optimization), and (5) decision tree. Performance measures, such as accuracy, sensitivity, and specificity with 10-fold cross-validation, were used for model comparisons. A smartphone app called CINV Risk Prediction Application was developed using the ResearchKit in iOS utilizing the decision tree algorithm, which conforms to the criteria of explainable, usable, and actionable artificial intelligence. The app was created using both the bulk questionnaire approach and the adaptive approach.

**Results:**

The decision tree performed well in both phases of high emetogenic chemotherapies, with a significant margin compared to the other algorithms. The accuracy measure for the six patient groups ranged from 79.3% to 94.8%. The app was developed using the results from the decision tree because of its consistent performance and simple, explainable nature. The bulk questionnaire approach asks 14 questions in the smartphone app, while the adaptive approach can determine questions based on the previous questions' answers. The adaptive approach saves time and can be beneficial when used at the point of care.

**Conclusions:**

This study solved a real clinical problem, and the solution can be used for personalized and precise evidence-based CINV management, leading to a better life quality for patients and reduced health care costs.

## Introduction

### Background

Chemotherapy is a drug treatment commonly used to treat nearly every type of cancer [1]. As estimated, each year, as many as 1 million Americans receive some type of chemotherapy [2]. Cancer cells multiply at an unusually faster rate compared to healthy cells, and chemotherapy is used to kill those fast-growing cells in the body. However, chemotherapy can lead to many side effects, such as nausea, vomiting, appetite changes, anemia, hair loss, constipation, and diarrhea, among others [3-11]. Chemotherapy-induced nausea and vomiting (CINV) are the two most frightful and unpleasant side effects of chemotherapy [3,4,12-15].

CINV can lead to consequences that affect both patients and the health care system as a whole. First, CINV engenders other side effects, such as nutritional deficits, dehydration, and electrolyte imbalance, which diminishes the quality of life in cancer patients [16-20]. Second, the various side effects of CINV lead to a low-quality social life [19,21]. Third, CINV can also lead to loss of workdays, which in return increases the economic burden [19,22-24]. Fourth, CINV surges health care costs arising from CINV-related outpatient visits, hospitalization, and the cost of drugs [18,19,22-27]. Fifth, intolerance of cancer patients toward CINV can lead to discontinuation of cancer treatment, leading to poor treatment outcomes, treatment failure, or even death [12,28-30].

The management of CINV is a complex process due to two factors. The first level of complexity arises from the different impacts of the different emetogenicity levels of the chemotherapeutic agents. The emetogenicity of chemotherapy is fractionated into four emetic risk categories based on the percentage of patients who suffer from CINV without antiemetics: (1) minimal (<10%), (2) low-emetogenic chemotherapy (LEC: 10%-30%), (3) moderate-emetogenic chemotherapy (MEC: 30%-90%), and (4) high-emetogenic chemotherapy (HEC: >90%). CINV has two different pathophysiological phases (acute and delayed) that can lead to different consequences, adding a second level of complexity. The acute phase of CINV occurs within the first 24 hours of chemotherapy. Chemotherapy triggers the release of serotonin in the peripheral pathway (gastrointestinal tract), which binds to the 5-hydroxytryptamine (5-HT_3_) receptors and sends a signal to the vomiting center in the medulla [31,32]. The central pathway is associated with the delayed phase of CINV that occurs after the first 24 hours of chemotherapy administration and may persist up to 1 week. This pathway is located in the brain, where chemotherapy triggers a neuropeptide release named substance P, which binds to the neurokinin-1 (NK-1) receptor in the vomiting center, causing CINV [31,32].

There are several antiemetic guidelines for the management of CINV, such as the American Society of Clinical Oncology (ASCO) guideline [33,34], the National Comprehensive Cancer Network (NCCN) guideline [35], and the guideline from the Multinational Association of Supportive Care in Cancer (MASCC) in cooperation with the European Society of Medical Oncology (ESMO) [36]. Despite the improvements in CINV management, many recent studies have reported various percentages of patients experiencing CINV with the use of antiemetics: 28% [37], 38%-52% [38], 56.1% [39], 61.2% [19], and 62% [20]. The guideline-recommended standard antiemetic prophylaxis takes only the chemotherapeutic emetogenicity into consideration for CINV management.

However, several patient-related risk factors can potentially worsen the risk of CINV, but none of the guidelines considers those factors [40]. Since physicians cannot entirely rely on the guidelines, they use their own experiences to manage CINV. Consequently, CINV management is inconsistent among physicians, since their decisions are subjective to their experiences in managing CINV [41].

The use of risk prediction algorithms for clinical decision making at the point of care would require completing and processing massive patient panels, which can be time consuming and can lead to inaccurate results [42]. In recent years, smartphones have become popular among physicians for accessing health care information at the point of care [43]. The advent of open-source frameworks, such as Apple ResearchKit, Apple CareKit, and Android frameworks (eg, PhoneGap), has opened up tremendous opportunities to capture patient-related data and deliver patient-specific clinical decision support information through smartphones. Data mining techniques are beneficial in predictive analytics on medical data [44]. Various machine learning (ML) algorithms have the potential to help build robust clinical decision support systems using clinical data. Smartphone apps integrated with robust clinical decision support developed from rigorously validated ML models and artificial intelligence (AI) can be immensely useful for clinicians and can significantly improve overall health care delivery.

### Objective

The objective of this study was to develop a smartphone app for clinical decision support to predict patients' risk of CINV using patient-related risk factors. ML algorithms, such as the decision tree, naive Bayes algorithm, logistic regression classifier, neural network, and support vector machine, were applied to determine the best-performing algorithm for CINV risk prediction based on electronic medical records (EMRs). Standard performance metrics, such as accuracy, sensitivity, and specificity, were used to compare the performance among the algorithms. This paper also illustrates the use of the ML model to develop a smartphone app and demonstrates its usage from the users' perspective. The developed app aims to help clinicians identify high-CINV-risk patients and can be integrated with antiemetic guidelines for better CINV management.

## Methods

### Data Sources and Population Selection

This was a retrospective study, and data were collected from the EMRs from a single center called the University of Missouri Ellis Fischel Cancer Center. The study was approved by the MU Health Sciences institutional review board. Our study included only patients who received chemotherapy and standard antiemetic prophylaxis (based on national antiemetic guidelines) at the oncology outpatient service from June 1, 2010, to July 31, 2012. However, we excluded patients with missing information and those who underwent concurrent radiotherapy or surgical procedures.

We planned to collect two independent data sets for each stage of CINV. Since acute and delayed CINV follows two different pathophysiologies, we planned to discover the patient-related risk factors for causing CINV during both phases independently. In each data set, there were three groups based on the emetogenicity level of the chemotherapy regimens. Of the four emetic risk categories, the minimal risk category of chemotherapy for causing CINV is not clinically crucial, since only less than 10% of those patients suffer from CINV. Thus, we collected data in three separate groups corresponding to three clinically meaningful categories: low, moderate, and high.

Our significant interest classes included both CINV and non-CINV cases. However, LEC led to CINV in less than 30% of patients, and the use of standard antiemetic treatment further reduced this percentage. Thus, the data set had few CINV cases compared to non-CINV cases. In addition, the number of CINV cases was higher than the non-CINV cases in the HEC group. Hence, class balancing in each data group (LEC, MEC, and HEC) was considered necessary. We addressed the class imbalance issue by making the data set's size in each class for each group approximately equal.

### Variable Selection

In a previous study, we completed a systematic review by following the Preferred Reporting Items for Systematic Reviews and Meta-Analyses (PRISMA) guideline to identify potential patient-related variables that cause CINV [45]. Our previous study used MEDLINE to identify articles that demonstrated patient-related risk factors of CINV through clinical studies. A total of 26 patient-related risk factors were documented in that study from reviewing 49 articles [46]. For this study, we included 14 independent variables and 1 dependent variable (CINV outcome) [46]. We chose the risk factors based on the recommendations from chemotherapy experts in the MU Elis Fischel Cancer Center and our literature review. The selected variables were also easy to collect through clinical encounters, which can facilitate the usability of the prediction model at the point of care before chemotherapy.

### Data Mining

Data mining or knowledge discovery in databases (KDD) can discover hidden patterns, previously unknown, and potentially useful information from data. In general, data mining algorithms are categorized into two groups: descriptive or unsupervised learning and predictive or supervised learning. In supervised learning, the class labels of the observations or tuples are known, whereas in unsupervised learning, those class labels are unknown. For this study, we developed a prediction model that falls into the supervised learning or classification category.

Classification is a supervised learning method for building classification models based on a data set (called training data) and the values in classifying attributes (called a class label). The classification model is used to predict the categorical class label. Classification is a two-step process in which the model is constructed in the first step and the accuracy of the model is determined using a data set (called test data set) in the second step. The accuracy of the classification model is the percentage of test data set tuples that are correctly classified by the model. To overcome the overfitting problem, the test data set must be independent of the training data set. In general, the classification model consists of IF-THEN rules or mathematical formulas. For our study, we used five popular data mining algorithms: (1) naive Bayes [47], (2) logistic regression classifier [48], (3) neural network (voted perceptron) [49], (4) support vector machine (using sequential minimal optimization) [50], and (e) decision tree [51-53]. There are several tools available for data mining. We used the most widely used tools, called WEKA [54]. Performance measures, such as accuracy, sensitivity, and specificity, were used for model comparisons. In addition, 10-fold cross-validation were used for model validation [55].

### Smartphone App Development

ResearchKit is an open-source framework based on iOS that makes it easy to create mobile apps. It allows researchers and drug developers to tailor it to their own particular needs, whether for collecting clinical research data, recruiting patients, or obtaining informed consent. The framework allows for collecting information through electronic data capture, creating a small task to gather any specific information required for the study, and then storing the data as part of a sandbox, thereby protecting patient information. We developed our smartphone app using some modules, including a survey engine, visual consent flow, and active tasks from this framework. As the users of this app will be care providers, and no identifiable data will be stored, we did not use the visual consent flow. The smartphone app was built using the algorithm that had the most consistent performance among the ML algorithms and is also explainable, usable, and actionable AI for clinical decision support.

## Results

### Data Summary

In total, 6124 records were extracted based on inclusion and exclusion criteria. The number of records was 3053 and 3071 for the acute-phase and the delayed-phase data set, respectively. Table 1 presents the frequency distribution of both data sets for combinations of three chemotherapy categories and two treatment outcomes.

**Table 1:** Data summary.

CINV^a^ treatment group			Records, n	CINV, n (%)		No CINV, n (%)
**Acute phase**						
	HECb^b^	1026		504 (49.12)	522 (50.88)	
	MEC^c^	1012		506 (50.00)	506 (50.00)	
	LEC^d^	1015		506 (50.15)	509 (49.85)	
	Total	3053		1519 (49.75)	1534 (50.25)	
**Delayed phase**						
	HEC	1166		586 (50.26)	580 (49.74)	
	MEC	891		447 (50.17)	444 (49.83)	
	LEC	1014		519 (51.18)	495 (48.82)	
	Total	3071		1552 (50.54)	1519 (49.46)	

^a^CINV: chemotherapy-induced nausea and vomiting.

^b^HEC: high-emetogenic chemotherapy.

^c^MEC: moderate-emetogenic chemotherapy.

^d^LEC: low-emetogenic chemotherapy.

### Data Mining Model Performance Comparison

**Figure 1:**
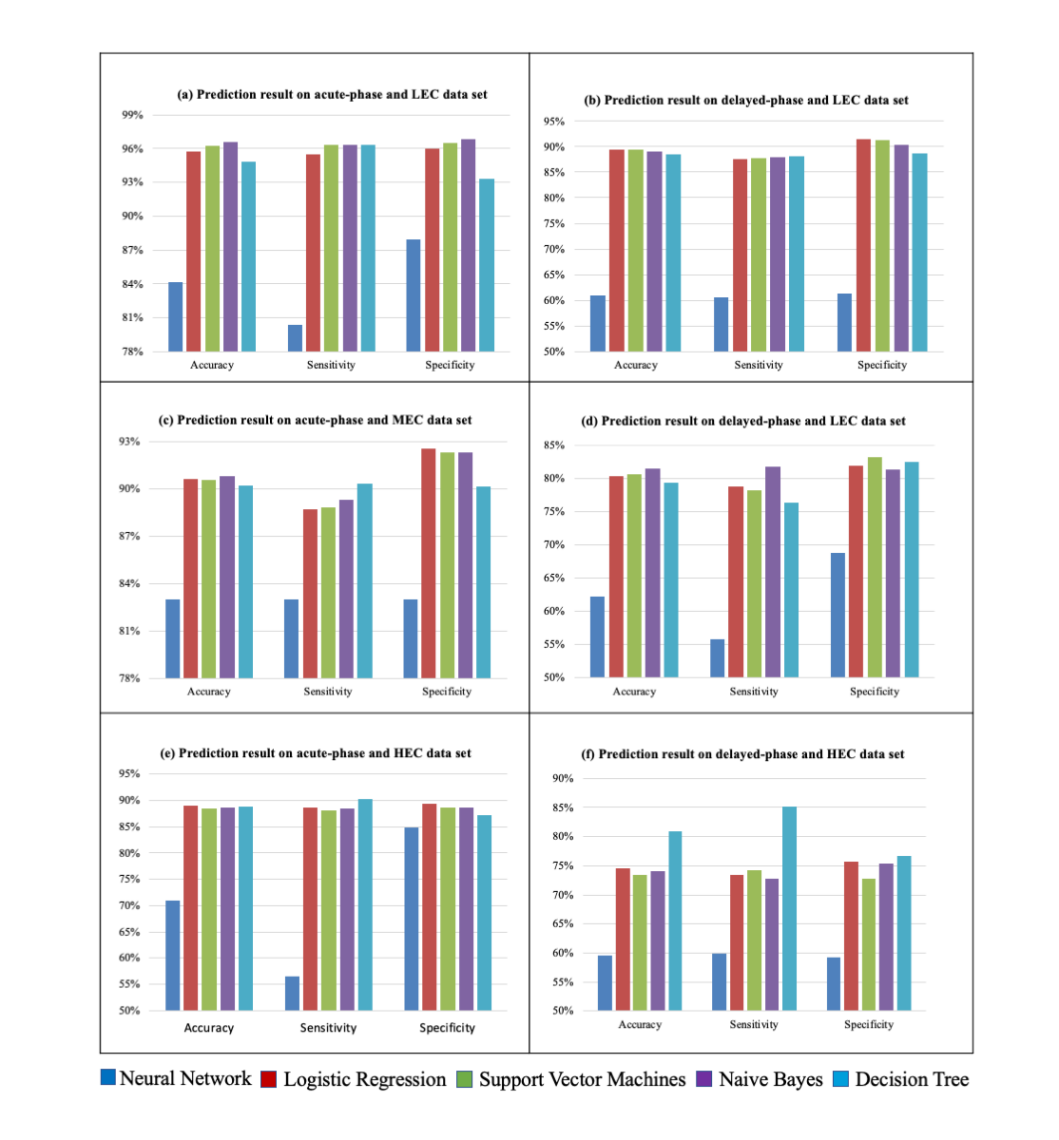
Accuracy, sensitivity, and specificity of different ML algorithms used to predict CINV status among patients. CINV: chemotherapy-induced nausea and vomiting; HEC: high-emetogenic chemotherapy; LEC: low-emetogenic chemotherapy; MEC: moderate-emetogenic chemotherapy; ML, machine learning.

The models' performances for all the emetogenicity levels and CINV phases (accuracy, sensitivity, specificity) are compared in Figure 1. The differences between performances of the different models were not consistent in each data set's model. The naive Bayes algorithm showed the best performance in the acute phase for LEC (the accuracy was 96.6%, sensitivity was 96.3%, and specificity was 96.8%), the acute phase for MEC (the accuracy was 90.8%, sensitivity was 89.3%, and specificity was 92.3%), and the delayed phase for MEC (the accuracy was 81.5%, sensitivity was 81.7%, and specificity was 81.3%). For the delayed phase for LEC, the support vector machine gave the best performance (the accuracy was 89.5%, sensitivity was 87.8%, and specificity was 91.3%).

The decision tree gave the most consistent performance in both phases of HEC, with a significant margin compared to the other algorithms. Although different algorithms gave the best performance for different stages, we selected the decision tree model to develop the app for its consistent performance across measures and its simple, explainable nature. Moreover, clinical decision support integrated with explainable, usable, and actionable AI is more convenient for oncologists to understand, and thus, it can help them understand the app's background functioning.

### Decision Tree Models

**Figure 2:**
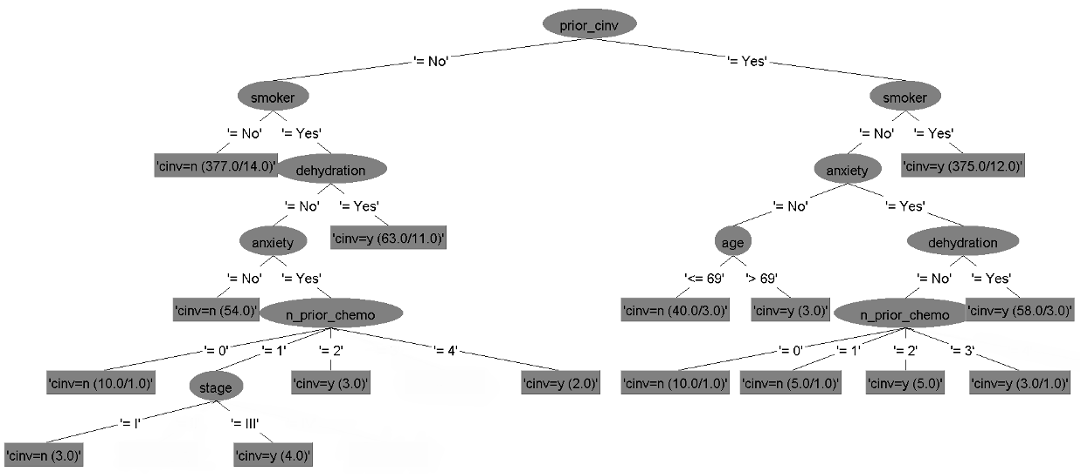
Decision tree. Phase: acute; emetogenicity: low. CINV: chemotherapy-induced nausea and vomiting.

**Figure 3:**
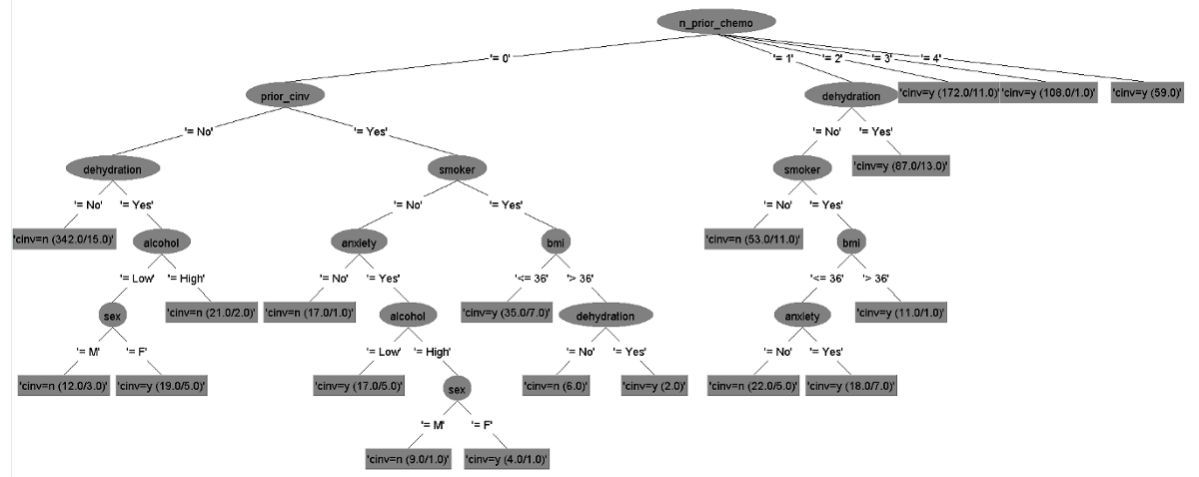
Decision tree. Phase: delayed; emetogenicity: low. BMI: body mass index; CINV: chemotherapy-induced nausea and vomiting.

**Figure 4:**
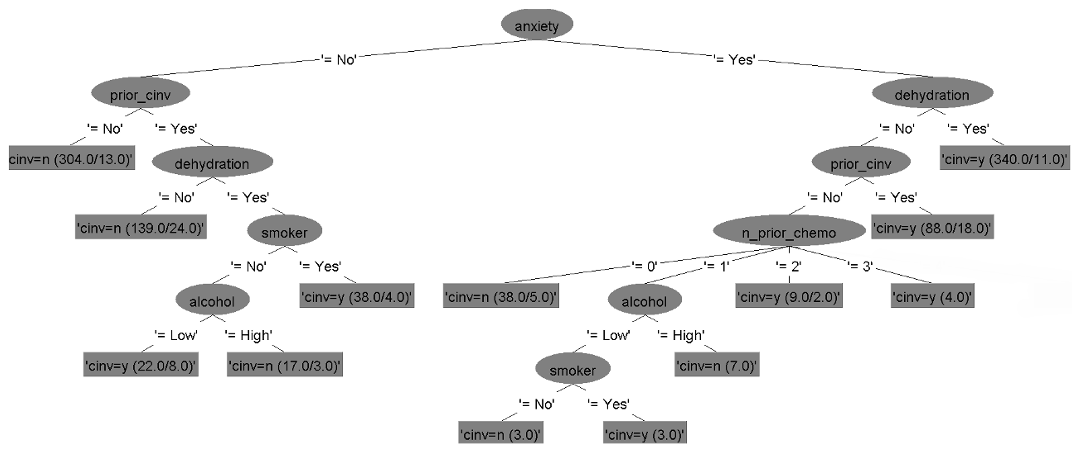
Decision tree. Phase: acute; emetogenicity: moderate. CINV: chemotherapy-induced nausea and vomiting.

**Figure 5:**
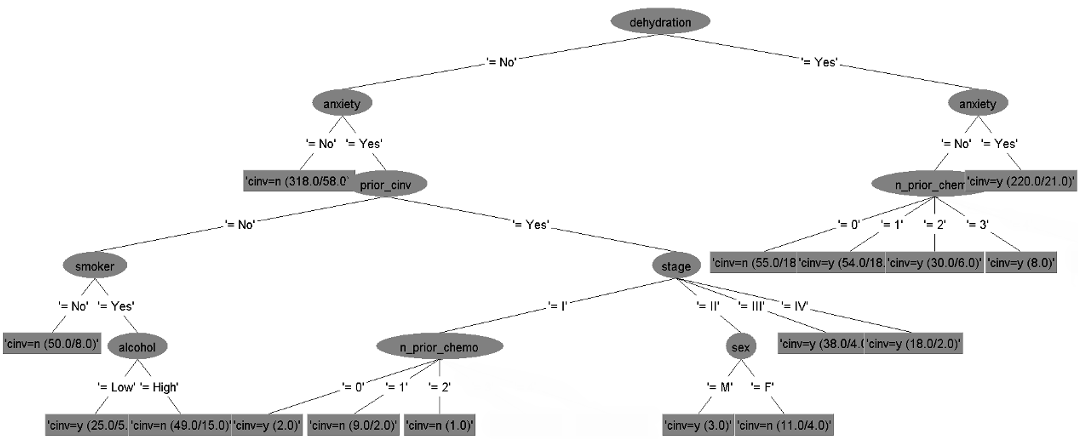
Decision tree. Phase: delayed; emetogenicity: moderate. CINV: chemotherapy-induced nausea and vomiting.

**Figure 6:**
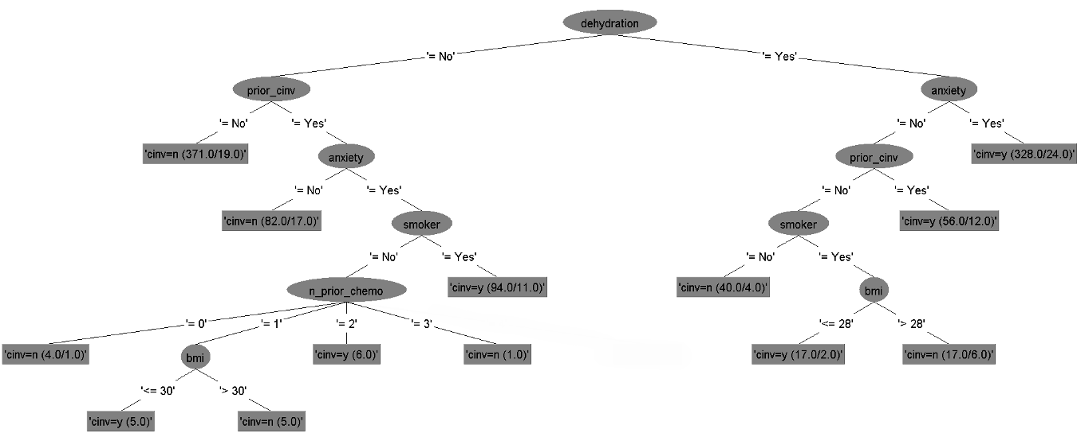
Decision tree. Phase: acute; emetogenicity: high. BMI: body mass index; CINV: chemotherapy-induced nausea and vomiting.

**Figure 7:**
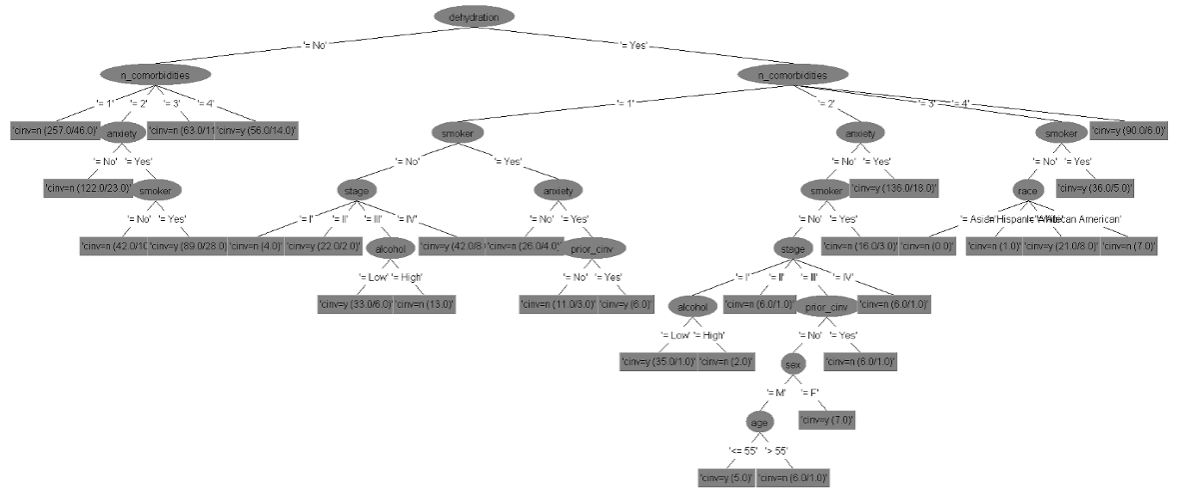
Decision tree. Phase: delayed; emetogenicity: high. CINV: chemotherapy-induced nausea and vomiting.

The six decision tree models for predicting CINV in both acute and delayed phases for each type of emetogenicity resulted in six flowcharts (Figures 2-7). Table 2 shows the description of the abbreviated form of each patient-related risk factor shown in the decision trees. We optimized the confidence factor for tree size and used the same confidence factor for all the decision trees. A threshold of >0 was used as the cutoff point. The accuracy of the six models was 94.8%, 88.5%, 90.2%, 79.3%, 88.7%, and 81%, respectively. In addition, sensitivity (correct prediction for the positive outcome of CINV) measures were 96.3%, 88.2%, 90.3%, 76.3%, 90.3%, and 85.2%, respectively, while specificity (correct prediction for the negative outcome of CINV) measures were 93.3%, 88.7%, 90.1%, 82.4%, 87.2%, and 76.7%, respectively.

**Table 2:** Patient-related risk factors and their abbreviations used in the decision trees.

Risk factor abbreviation	Description
smoker	Is the patient a current smoker?
race	Race of the patient
age	Age of the patient in years
bmi	Body mass index during chemotherapy
anxiety	Did the patient have anxiety during chemotherapy?
prior_cinv	History of previous CINV^a^
n_prior_chemo	Number of prior chemotherapy regimen
n_comorbidities	Number of comorbidities
sex	Sex of the patient
alcohol	Alcohol consumption
stage	Stage of cancer
type	Type of cancer
dehydration	Did the patient have dehydration during chemotherapy?

^a^CINV: chemotherapy-induced nausea and vomiting.

### Clinical Decision Support Smartphone App

The clinical decision support smartphone app for CINV was developed using the output of the decision tree models obtained from the above analyses. The app was built on iOS and developed considering space usage and the possible variation of its users' technological skills. We created active tasks, depending on the flowcharts. In addition, the survey engine helped us to easily implement the questionnaire survey.

**Figure 8:**
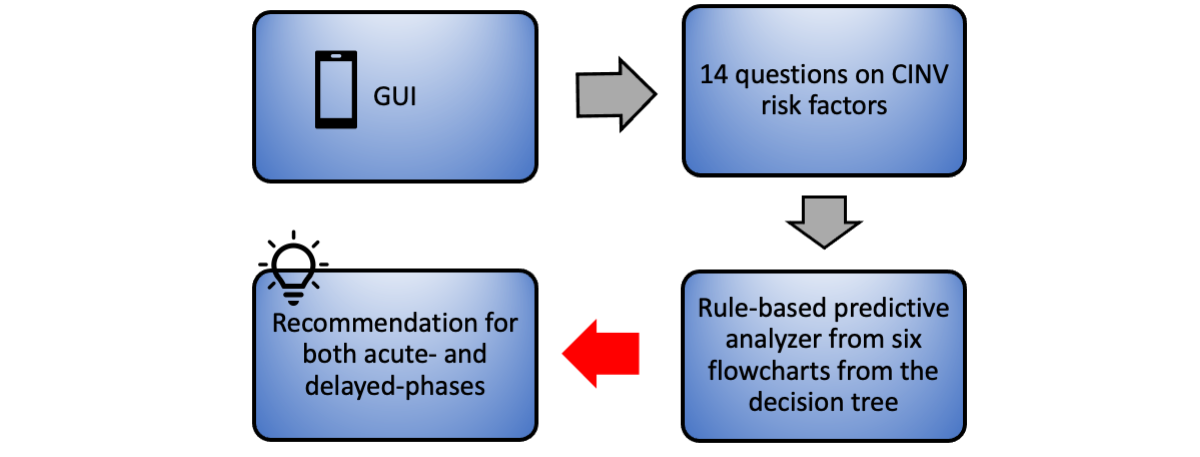
Flow diagram of CINV risk prediction smartphone app using the bulk questionnaire approach. CINV: chemotherapy-induced nausea and vomiting; GUI: graphical user interface.

**Figure 9:**
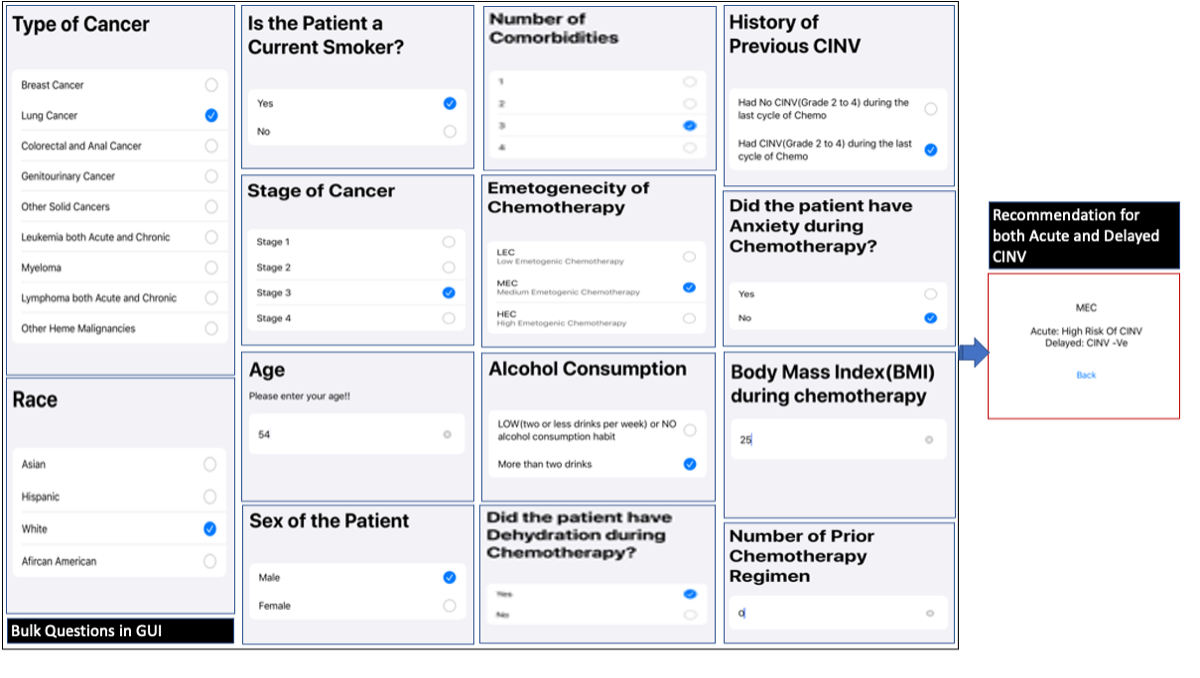
Application GUI for the bulk questionnaire approach. CINV: chemotherapy-induced nausea and vomiting; GUI: graphical user interface.

The app was created using two different approaches: (1) the bulk questionnaire approach and (2) the adaptive questionnaire approach. In the bulk questionnaire approach, all 14 questions regarding CINV risk factors were asked one by one. After receiving the responses of the patients on all the questions, the predictive analyzer predicted the recommendations on both phases, depending on the six flowcharts obtained by applying the decision tree algorithm. In Figure 8, the flow for the bulk approach is shown. For a better experience, the clinician has the freedom to go back and change the input and recalculate the answer. An example of a set of answers is given in Figure 9. Depending on all the answers and using the six flowcharts' logic, the system selects the result for both the acute and delayed phases and displays it.

The main limitation of the bulk questionnaire approach is that the physician at the point of care has to answer all the 14 questions to get to the final recommendation, even though not all questions are required for decision making for that patient. ResearchKit allows us to customize the questionnaire by adding features such as skipping questions or creating multiple paths, depending on the answer of the parent node of the decision trees. However, in this study, risk factors did not form a consistent hierarchy across the flowcharts, and thus skipping questions from a fixed questionnaire did not help. Moreover, some of the flowcharts had the same child under the parent node regardless of the answer, following different paths afterward. For instance, in Figure 5, the parent node is dehydration but the child node is anxiety regardless of whether dehydration is true or false. This motivated us to build a more time-energy-efficient approach called the adaptive questionnaire approach.

**Figure 10:**
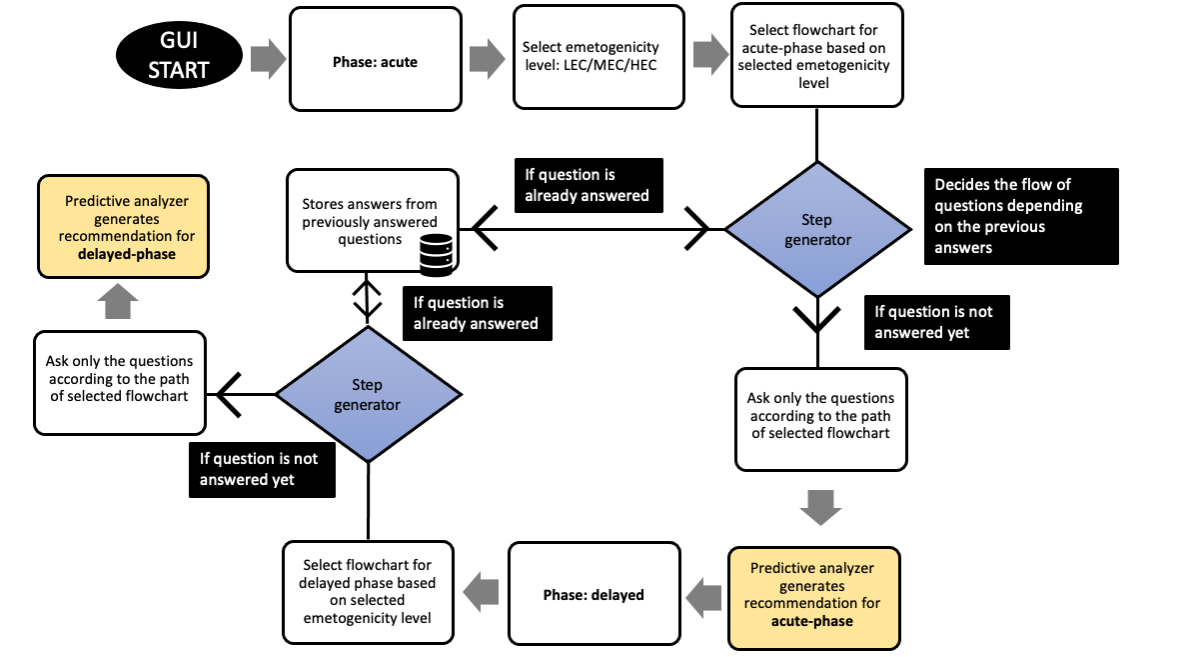
Flow diagram of CINV risk prediction smartphone app using the adaptive questionnaire approach. CINV: chemotherapy-induced nausea and vomiting; GUI: graphical user interface; HEC: high-emetogenic chemotherapy; LEC: low-emetogenic chemotherapy; MEC: moderate-emetogenic chemotherapy.

In an adaptive approach, the rule-based system first chooses a flowchart for the acute phase, depending on the emetogenicity level. A flowchart can have different paths, depending on the answers to the question as they come in the hierarchy of the decision tree. This approach follows a single path from the flowchart to generate a questionnaire for the clinician and saves all the answers in a database. Upon recommending the acute phase, the rule-based system chooses another flowchart for the delayed phase. This time, not all the questions in that flowchart are asked; instead, the app asks only the unanswered questions. There is a step generator feature at play for both acute and delayed phase prediction. The step generator determines the question paths for the patient, generates a new step if the question is unanswered, and use the answer from the saved answers for the already answered questions to generate the recommendation. In this approach, only the minimum questions needed to give a recommendation are included in the questionnaire, making the app more effective, faster, and user friendly. In Figure 10, the flow for the adaptive survey approach is shown.

**Figure 11:**
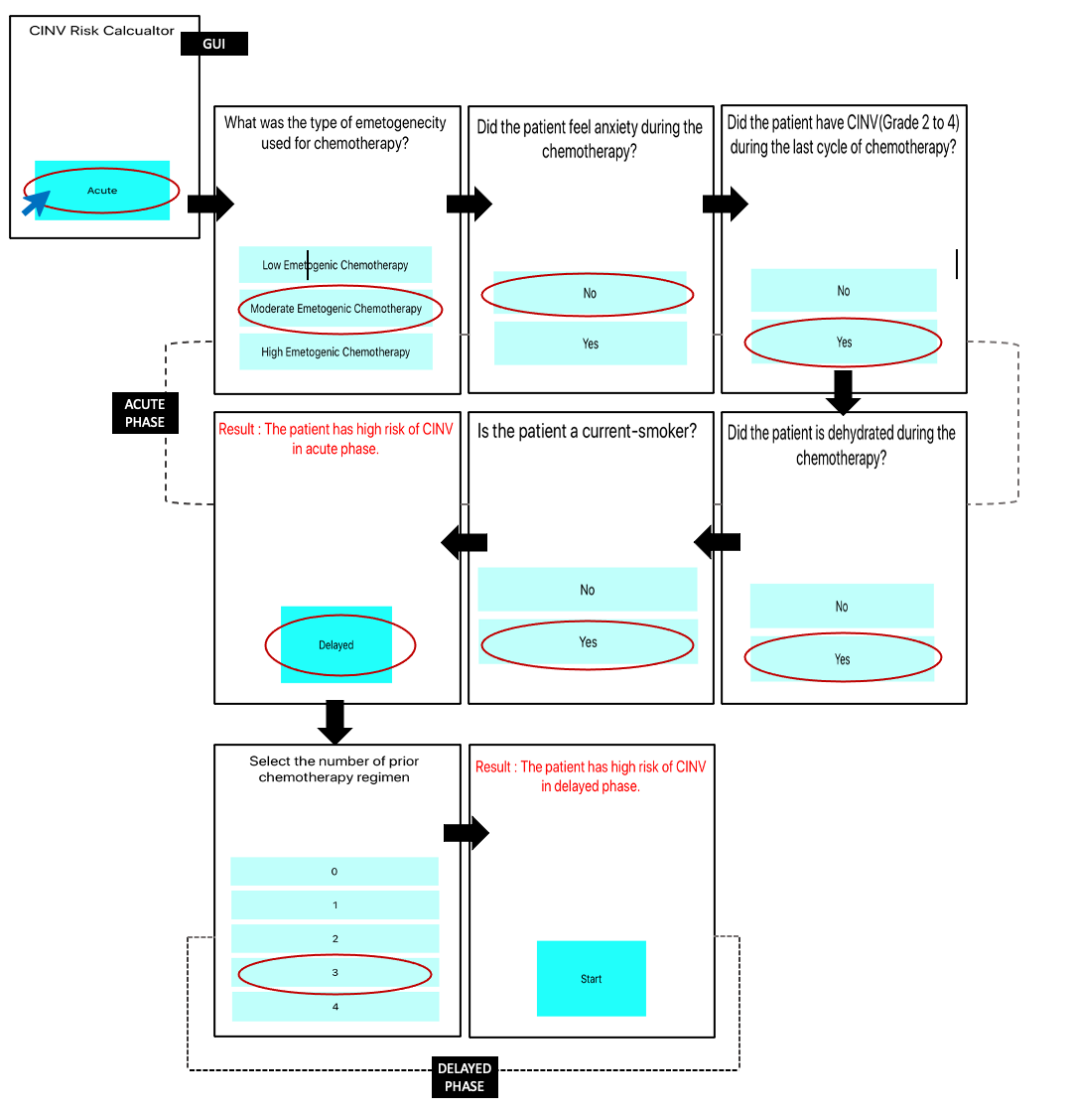
Application GUI for the adaptive questionnaire approach. CINV: chemotherapy-induced nausea and vomiting; GUI: graphical user interface.

For the adaptive approach, the app's questionnaire comes in dynamic format. The flow of the adaptive approach for a single path is illustrated in Figure 11. In this scenario, the user selected MEC as the emetogenicity of chemotherapy for the acute phase, and the model chose the decision tree for acute MEC shown in Figure 4. According to this flowchart, the first question was “whether the patient had anxiety during the chemotherapy,” for which the user selected ”no“ as an answer. Following this answer, the next question was ”the history of previous chemotherapy.” The user selected “yes,” which led to the next question about ”dehydration.” Since the answer was “yes” for dehydration, the next question was about “smoking status.” Only by asking these four questions, the system identified that the patient is at high risk of CINV. Although there are 14 risk factors, our dynamic approach only asks the questions that are necessary, choosing one pathway from the flowchart, which depends on the answers to the previous questions.

The user started back again and now selected the delayed phase in the app for determining CINV risk. The system selected the flowchart from Figure 5 this time. One advantage of this adaptive approach is that it will not ask questions that have already been answered. For example, although in the delayed-phase flowchart, the first question was about dehydration, this was not asked, since this was already answered in the acute-phase mode. The question of anxiety was also skipped for the same reason. The third question in the delayed-phase flowchart was about ”the number of prior chemotherapy regimens.” Since this question was never asked, the system picked this question next and the user selected 3 as an answer. Thus, using the answers to these questions, the app generated the recommendation that the patient has a high risk of CINV in the delayed phase.

## Discussion

### Principal Findings

CINV is a major side effect of chemotherapy among cancer patients. Appropriate examination of patient-specific risk factors before selecting premedications for CINV is critical in cancer care [56]. Better control of CINV has both short- and long-term effects in cancer care, leading to improved therapy tolerability, less anxiety, higher patient satisfaction, and avoidance of immediate discontinuation of the treatment [28,57-59]. Our previous study on finding risk factors through a systematic literature review shed light on the prevalent risk factors of CINV, as seen in the existing literature [46]. Patient-specific factors, such as smoking and alcohol status, sex, age, and the body mass index (BMI), can play a vital role in determining their effect on CINV. This study used data mining to discover significant relationships among the patient-related risk factors that influence the occurrence of CINV. Six independent data sets (three chemotherapy groups and two phases of CINV in each chemotherapy group) were individually analyzed to build the best-possible prediction models for CINV prediction. The risk factors used for building the models can be easily collected at the point of care or are available in the hospital EMRs. Among the popular data mining algorithms used for our study, the decision tree model performed consistently across the measures for both CINV phases.

A rule-based app can be considered an appropriate choice for its simplicity in explaining the model to a clinician and implementing it in a software application. Thus, we developed a CINV smartphone app using the results from the decision tree model because of its consistent performance and simplicity. We implemented two approaches, bulk and adaptive, to develop the CINV risk prediction app using ResearchKit. If the questions could be generated from multiple flowcharts, designing a fixed-order questionnaire might not help build an efficient app. The question hierarchy was not consistent across different tree models. Instead of asking input to all variables, we developed an adaptive approach to present a minimal number of questions for computing the prediction. The fixed (bulk)-order approach will ask 14 questions for any of those 115 decision paths, but for the adaptive approach, the maximum questions asked will be equal to the depth of that flowchart (up to 9 questions). This makes the app both time and energy efficient for the user and can reduce the physicians' time at the point of care.

The developed smartphone app for recommending patients at risk of CINV can help improve the prevention of CINV among cancer patients. The target users (ie, clinicians) can use this app at the point of care during the prescription of antiemetics. This app will help identify patients at risk of CINV based on patient-related risk factors. Having this knowledge of the patients before the prescription of antiemetics can help design a better treatment plan, leading to better CINV management. Furthermore, the app took significantly less space and was developed considering the possible variation in users' technological skills. It does not require any permission, which will help users use it more effortlessly. The oncologist will have complete access to the risk calculation algorithm in their smartphone, which will drastically reduce the amount of time required to help a large group of people and will have the flexibility to provide personalized care to every patient, improving their quality of life.

### Limitations

In this study, the data were collected by retrospective record review. Prospective validation is needed to confirm the usefulness of the model in a real clinical setting. The research also shows that female patients with pregnancy-related nausea and vomiting have a higher risk of CINV. However, this information was missing from our data set. This information could considerably enhance the prediction results. The data have a lower representation of Asians and Hispanics. A multicenter or multinational study, including various populations, is needed to overcome this shortcoming. In addition, if we use EMR data to integrate with the app, there is no difference between the bulk and adaptive approaches. However, if the app is used as a prediction tool at the point of care, the adaptive approach is more time and energy efficient, thus decreasing the chances of wrong input answers. In addition, for hospitals without any EMR system, this app can be extremely beneficial for cancer patients.

### Future Work

In the future, our plan is to deploy this app in point-of-care settings by integrating it into EMRs to predict the risk of CINV. We can also perform a clinical study for estimating outcomes and improvement. Currently, this app is developed only for the iOS platform, which can be expanded to Android in the future.

### Conclusions

This study aimed to solve a real clinical problem, and the solution can reduce the gap between clinical practice and evidence-based guidelines for CINV management. Our study will promote the notion of precision medicine by integrating patient-related risk factors and antiemetic treatment recommendations. Hence, our efforts can lead to increased quality of the patients' life and reduced health care cost. An effort to reduce the care provider's time has high importance at the point of care. A less time-consuming decision support tool to predict patients at risk will help care providers provide better care in general.

## Abbreviations

AI: artificial intelligence
ASCO: American Society of Clinical Oncology
BMI: body mass index
CINV: chemotherapy-induced nausea and vomiting
EMR: electronic medical record
ESMO: European Society of Medical Oncology
GUI: graphical user interface
HEC: high-emetogenic chemotherapy
KDD: knowledge discovery in databases
LEC: low-emetogenic chemotherapy
MASCC: Multinational Association of Supportive Care in Cancer
MEC: moderate-emetogenic chemotherapy
ML: machine learning
NCCN: National Comprehensive Cancer Network
NK-1: neurokinin-1
